# Extraocular sebaceous carcinoma: tumor presentation of rapid evolution^☆^

**DOI:** 10.1016/j.abd.2020.09.017

**Published:** 2022-01-15

**Authors:** Luana Moraes Campos, Joana Alexandria Ferreira Dias, Paula Basso Lima, Sílvio Alencar Marques

**Affiliations:** Faculdade de Medicina, Universidade Estadual Paulista, Botucatu, SP, Brazil

Dear Editor,

Sebaceous carcinoma (SC) is a rare malignant neoplasm derived from the adnexal epithelium of the sebaceous glands, with a higher incidence in the ocular region, particularly in the eyelid region, and has a potentially aggressive behavior.^1^, ^2^, ^3^ Older age, previous radiotherapy, and association with Muir-Torre syndrome are predisposing conditions.^1^, ^2^, ^3^

This is the case report of a 75-year-old white male patient with a history of squamous cell carcinoma (SCC), referred for treatment of facial lesion noted three months before, with rapid growth and bleeding episodes associated with trauma. Upon examination, a 5-cm, rounded, erythematous-violaceous, pre-auricular tumor was observed, with friable and necrotic areas associated with a 1-cm satellite lesion with similar characteristics and a post-SCC excision skin graft scar (Fig. 1). No regional lymph node enlargement was detected. The hypotheses were SCC, SCC metastasis, and angiosarcoma. Histopathological examination (Figure 2, Figure 3) showed a dermal neoplasm with polygonal clear cells, evident nuclear pleomorphism, cell debris, and frequent mitoses. Immunohistochemistry disclosed positivity for epithelial markers AE1/AE3 and epithelial membrane antigen (EMA) which, associated with histopathological findings, allowed the diagnosis of sebaceous carcinoma (SC), and thus, the patient was referred to the Head and Neck Surgery Division of the institution.

**Figure 1:**
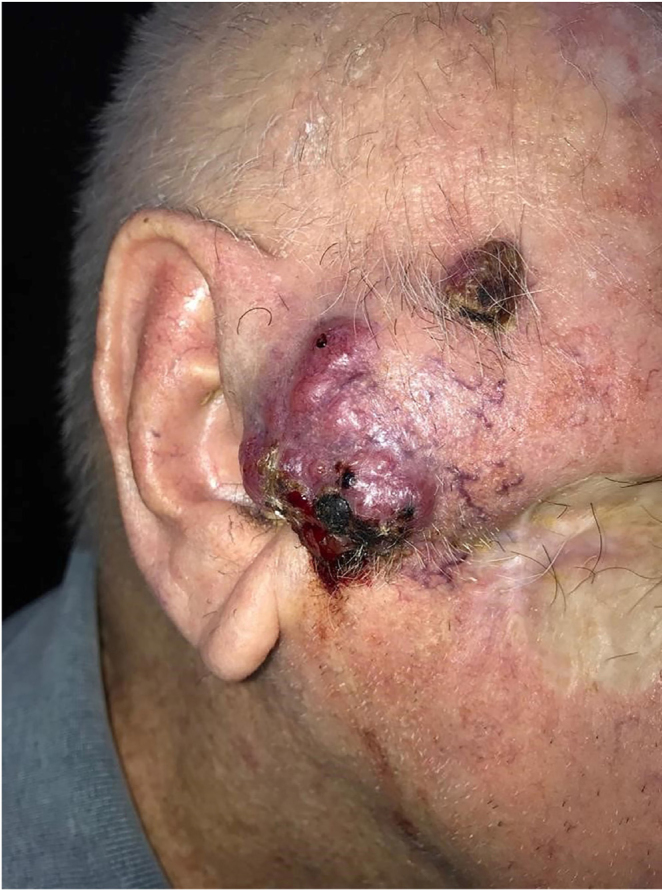
Extraocular sebaceous carcinoma. Tumor measuring 5 cm, rounded, with an erythematous-violaceous color, located on the right preauricular region, with friable and necrotic areas. Satellite lesion measuring 1 cm with similar features besides a SCC excision scar.

**Figure 2:**
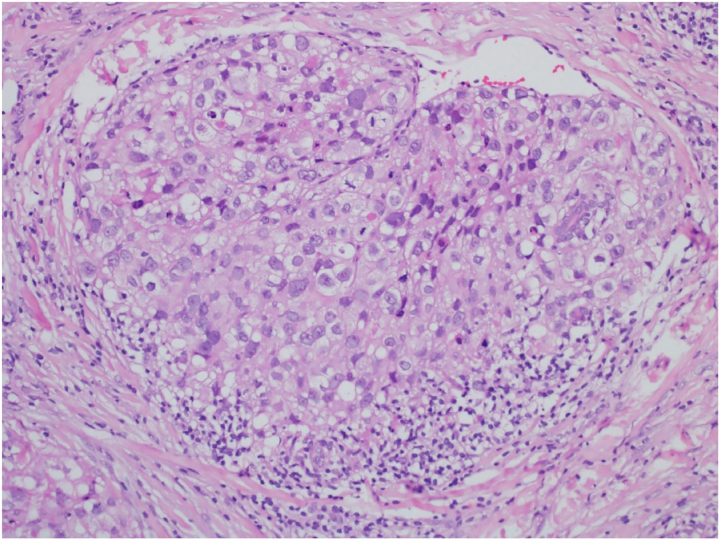
Extraocular sebaceous carcinoma. Neoplasm characterized by polygonal clear cells, nuclear pleomorphism, cell debris, and frequent mitoses (Hematoxylin & eosin, ×40).

**Figure 3:**
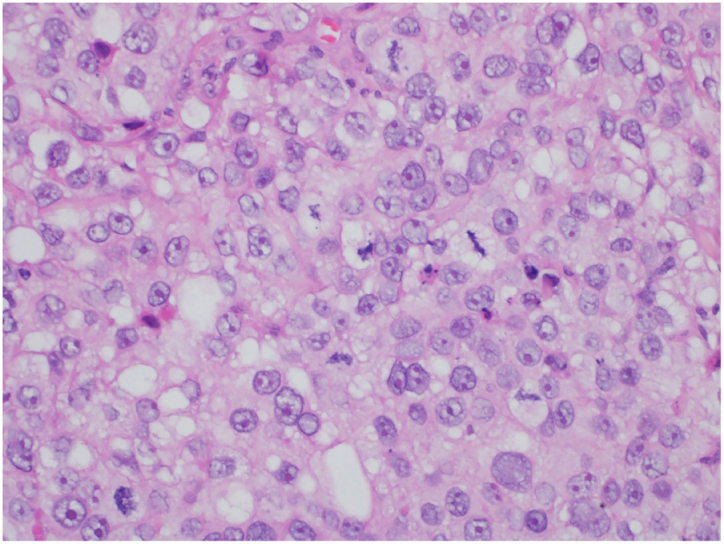
Extraocular sebaceous carcinoma. Detail of neoplastic cells showing nuclear pleomorphism, prominent nucleoli, and multilobular cytoplasm (Hematoxylin & eosin, ×400).

In a review of 1349 SC cases, a predominance of males (54%) was observed, as well as mean age of 73 years, 86% whites, 38.7% on the palpebra, with a survival rate of 91.9%, and 79.2% in 5 and 10 years respectively.^1^ The most frequent metastases were found in the lymph nodes.^1^, ^3^ Most cases occur *de novo*, although it may originate from benign sebaceous lesions and, when located in the upper or lower eyelid, it is associated with the Meibomian and Zeis glands.^1^, ^2^, ^3^ Clinical presentation is variable; it is usually painless and slow-growing, but it can be rapid-growing and aggressive.^2^

It is the third or fourth most frequent malignant neoplasm of the eyelids, depending on the reference.^1^, ^2^, ^3^ The most frequent extraocular location is the cephalic segment, especially the face. The diagnosis of SC should be a warning sign, as it is a possible marker of Muir-Torre syndrome, a genodermatosis characterized by the presence of skin tumors of sebaceous origin associated with systemic malignancies, particularly of the gastrointestinal tract.^1^ A subcutaneous nodule is usually observed in SC, which is normochromic; however, it may disclose different morphologies, colors and behavior, depending on its place of origin.^1^, ^2^, ^3^

The differential diagnosis of extraocular SC includes basal cell carcinoma (BCC), SCC, amelanotic melanoma, Merkel cell carcinoma, and cutaneous lymphoma.^1^, ^3^ The immunohistochemical use of markers for BerEP4, EMA (negative in BCC), AE1 and AE3 (negative in melanoma, lymphomas), adipophilin (negative in SCC, Merkel), p53 and Ki-67, will aid in the diagnosis and prognosis.^1^, ^2^, ^3^, ^4^ The treatment comprises surgical resection with a 1-cm margin or the use of the micrographic surgical technique.^2^ A therapeutic option in cases of metastatic SC to the lungs and CNS is immunotherapy with pembrolizumab, which belongs to the class of inhibitors of anti-PD1 immunological checkpoints (programmed death-1), and is also used in metastatic melanoma and Merkel cell carcinoma.^5^

This case report exemplifies a case of an atypical presentation of extraocular SC, especially due to the rapid growth and aggressiveness of the tumor, which when diagnosed had a specific satellite lesion and tumorous clinical aspect.

## Financial support

None declared.

## Authors’ contributions

Luana Moraes Campos: Approval of the final version of the manuscript; design and planning of the study; critical review of the literature.

Joana Alexandria Ferreira Dias: Approval of the final version of the manuscript; critical review of the manuscript.

Paula Basso Lima: Approval of the final version of the manuscript; collection, analysis, and interpretation of data; critical review of the manuscript.

Sílvio Alencar Marques: Approval of the final version of the manuscript; drafting and editing of the manuscript; critical review of the literature; critical review of the manuscript.

## Conflicts of interest

None declared.
